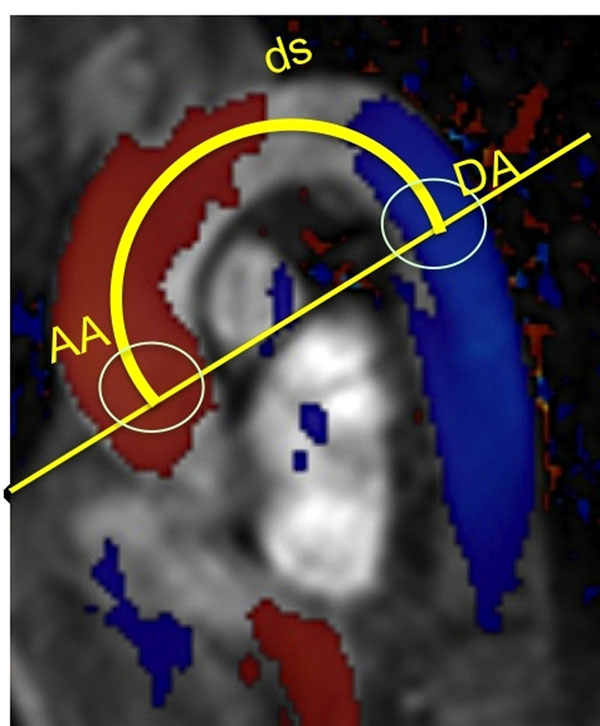# Gender differences in pulse wave velocity in young healthy adults at rest and exercise - the WellHeart Study

**DOI:** 10.1186/1532-429X-15-S1-E83

**Published:** 2013-01-30

**Authors:** Valentina O Puntmann, Kaleab N Asrress, Michael Marber, Simon Redwood, Sven Plein, Eike Nagel

**Affiliations:** 1 Cardiovascular Imaging, King's College London, London, UK; 2 Cardiovascular Division, King's College London, London, UK

## Background

Elderly women have increased aortic stiffness, measured by pulse wave velocity (PWV), and show little increase in PWV with inotropic stress. The aim of this study was to examine gender related differences in aortic stiffness at rest and during physiological exercise stress in young non-athletic subjects.

## Methods

Eighteen healthy subjects without known cardiovascular disease (mean age 28 years; male =10, all non-smokers) underwent cardiovascular magnetic resonance (CMR) imaging at rest and supine bicycle exercise for cine and high-resolution flow imaging.

## Results

At rest both genders demonstrated similar haemodynamic parameters and PWV. To achieve 85% of the age-predicted heart rate (apHR), men required significantly greater workload (p=0.05) and showed higher systolic blood pressure (BP, p=0.03) than women. Imaging at 60% apHR, sustained by hand-grip exercise, revealed an increase in stroke volume and cardiac index in men (p=0.05), whereas women showed no difference from rest (p=0.45). Men showed a strong increase in PWV (p=0.02), whereas women showed only a trend towards a difference (p=0.09).

## Conclusions

In young healthy non-athletic males there is a greater increase in PWV and systolic BP than women. Our findings concord with previous observations in elderly population during inotropic stress and inform on the gender differences in maximal performance and ageing.

## Funding

NIHR

**Figure 1:**